# Recruitment of family caregivers of persons with dementia: Lessons learned from a pilot randomized controlled trial

**DOI:** 10.3389/fpain.2023.1125914

**Published:** 2023-03-27

**Authors:** Sama Joshi, Taeyoung Park, Lilla Brody, Kiana Cruz, Priya Mukhi, M. Carrington Reid, Keela Herr, Karl Pillemer, Catherine Riffin

**Affiliations:** ^1^ Department of Medicine, Weill Cornell Medicine, New York, NY, United States; ^2^ Hackensack Meridian School of Medicine, Seton Hall University, South Orange, NJ, United States; ^3^ College of Human Ecology, Cornell University, Ithaca, NY, United States; ^4^ College of Nursing, The University of Iowa, Iowa City, IA, United States

**Keywords:** caregiving, pain, dementia, recruitment, intervention, method, enrollment, randomized controlled trial

## Abstract

Family caregivers play an essential role in supporting the health and well-being of older adults with dementia, a population projected to increase rapidly over the coming decades. Enrolling caregivers of people with dementia (PWD) in research studies is vital to generating the evidence necessary to support broader implementation of efficacious intervention programs in real-world care delivery, but a range of challenges impede recruitment and enrollment of sufficiently large and representative sample sizes. In this article, we characterize the challenges and lessons learned from recruiting caregivers of PWD to participate in a pilot randomized control trial. We utilize Bronfenbrenner's ecological model to categorize the challenges into three levels: *individual* (i.e., understanding caregivers’ time constraints and motivations), *community* (i.e., reaching underrepresented populations and accessing caregiver support groups) and *institutional* (i.e., obtaining informed consent and navigating research registries). We found that establishing rapport and maintaining flexibility with participants was crucial for motivating individuals to enroll in our study. Building trust with local communities by collaborating with support group leaders, appointing a co-investigator who is already embedded within a given community, and establishing equitable partnerships with organizations increased recruitment rates. At the institutional level, engaging experts in regulatory affairs and geriatrics may help overcome barriers in obtaining approval from institutional review boards. We also recommend using research registries of individuals who offer their contact information to researchers. The lessons learned from our research—including the challenges and potential solutions to overcome them—may promote more effective and efficient recruitment in future research.

## Introduction

In 2020, more than 7 million Americans aged 65 and older had some form of Alzheimer's disease or dementia. This number is projected to nearly double by 2040 if similar demographic and health trends persist (1). Such trends have significant implications for the U.S. healthcare system and for families who provide longitudinal dementia care (2). From 2015 to 2020, the number of dementia caregivers in the U.S rose from 10.5 million to 12.5 million (3). These unpaid caregivers play crucial roles in helping older persons with dementia (PWD) with self-care, mobility, and household activities (4), but they commonly suffer from emotional and physical hardships as well as unmet needs for training and support (5–7).

Interventions to support family caregivers of PWD are critical to enhancing quality of life and health outcomes of both caregivers and PWD. Yet, despite increasing federal investments in dementia care research (8, 9), notable deficits in the intervention literature remain (10). To date, clinical trials of caregiver interventions have suffered from small sample sizes with limited power to detect treatment benefits and to draw conclusions about a study's effectiveness (11, 12). With rare exceptions, interventions have relied on racially, ethnically, and culturally homogeneous samples (13, 36).

Addressing these methodological shortcomings is contingent upon successful recruitment and enrollment of caregiver participants, but major barriers impede investigators from effectively enrolling caregivers in clinical trials. These barriers encompass sociocultural issues reaching traditionally marginalized groups (14), high costs associated with robust recruitment strategies (11, 15), and caregivers’ own time and mobility constraints (11, 16). Identifying actionable strategies to overcoming these challenges is critical to supporting future research that effectively recruits and enrolls sufficiently large, representative samples, which is necessary to generate an evidence base that supports broadscale implementation and dissemination of efficacious intervention programs.

Researchers at Weill Cornell Medicine and the University of Iowa School of Nursing conducted a pilot randomized controlled trial (RCT) evaluating the Pain Identification and Communication Toolkit (PICT). The goal of this article is to characterize the challenges our research team encountered and lessons we learned from recruiting and enrolling family caregivers of PWD in this RCT.

### Parent study

We present key elements of study design and methods used to recruit prospective participants in the PICT pilot RCT. PICT is a multicomponent, manualized intervention designed to help caregivers recognize and communicate about pain in their care recipients (17). Caregivers randomized to PICT received 4 sessions over the phone by a trained social worker. The control condition consisted of an information pamphlet about pain in dementia. All participants completed baseline and 3-month follow-up assessments and were compensated for their participation. The PICT program manual is available upon request.

Recruitment for this study involved (1) presentations to dementia caregiver support groups, (2) direct outreach at ambulatory care practices serving older adults with dementia, (3) study flyer postings in the community (e.g., senior centers, grocery stores), (4) informational posts on online research registries, listservs, and social media outlets, and (5) word-of-mouth. Recruitment strategies were transitioned fully online at the onset of the COVID-19 pandemic. The study period was March 2019, to August 2021.

Overall, we achieved a high response rate among prospective study participants: Of the 155 individuals who were initially approached for our study, only 33 declined to participate, suggesting that the strategies we employed were effective in overcoming recruitment challenges. Out of the 155 initially approached, 122 were assessed for eligibility and 88 were excluded, resulting in 34 participants in the PICT pilot RCT. Before the transition to virtual recruitment in March 2020, 40 participants were approached and 17 were enrolled while 115 participants were approached and 17 were enrolled after this shift. Our final sample was composed of participants from diverse racial and ethnic backgrounds: 73% White, 10% Black, 7% Asian, 10% multiracial; 10% Hispanic.

## Barriers to recruitment and potential solutions

In this section, using Bronfenbrenner's ecological theory as a guiding framework (18), we classify recruitment challenges and potential solutions into three broad domains: individual (microsystem), community (mesosystem and exosystem), and institution (macrosystem). This organizational structure serves as a roadmap for this paper and is intended to help researchers overcome recruitment challenges at a given level.

Community- and institution-level barriers were identified by reviewing our study's recruitment patterns and enrollment data. Individual-level challenges were identified during exit interviews with study participants. We contextualized patterns in each of these domains within the current literature on recruitment barriers faced by other researchers.

### Individual-level barriers

Common challenges to recruiting family caregivers into research studies include caregivers’ time constraints and motivating caregivers to take part in research. Below, we describe our approaches to overcoming these individual-level barriers.

#### Challenge: Caregivers’ time constraints

Lack of time was frequently cited by eligible persons who declined to enroll in the trial or chose to refer the study to other caregivers in their network. As one caregiver noted: “I have so much on my plate that I need to do”. This challenge was exacerbated by the COVID-19 pandemic. Caregivers, especially those who co-resided with their care recipients, reported devoting considerably more time to caregiving during the pandemic (19, 20). Throughout our recruitment process, many caregivers expressed difficulty balancing caregiving with other responsibilities. Enrolling caregivers with high levels of stress was especially challenging. This experience is consistent with recent data showing that recruitment and enrollment of caregivers varies according to their stress level (21).

#### Solutions

We used a two-pronged strategy to minimize the time burden on caregivers. First, we communicated succinctly about the study. Creating promotional materials that were easy-to-read and simply worded not only minimized caregivers’ mental load but also increased participation of individuals with varying levels of health literacy, thus building a more representative sample (22). Second, we made efforts to accommodate participants’ preferences regarding the timing and mode of data collection by noting their preferred method of correspondence early in the enrollment process and remaining flexible about rescheduling sessions. To justify the time commitment for the study, we emphasized our high retention rate and positive feedback about intervention when recruiting additional participants.

#### Challenge: Motivating caregivers to participate in research

Given the heavy burden associated with caring for PWD and the multiple responsibilities of family caregivers, this population requires greater incentives to participate in research (23).

#### Solutions

Our recruitment strategies focused on three areas identified by prior literature to be associated with increased motivation among caregivers to engage in research: (1) receiving education for their caregiving role, (2) making a difference to others, and (3) forming personal connections with researchers (24). For example, a caregiver cited their desire to receive education about effective caregiving as a motivator for participating in our research study: “I've been a caregiver, you know, for several years now, but… there's always something else [I] can learn… that's one reason I was really interested in [participating].” We emphasized the possible individual-level benefits to participation, underscoring that our intervention could help caregivers improve their abilities to identify and communicate about pain and that our intervention was applicable across the spectrum of pain and dementia severity.

With respect to making a difference to others, prior research has found that altruism drives participation in research more than financial compensation (25). We therefore framed our study in the broader context of how it would make a difference to caregiver populations in the future and accepted participants’ requests to be updated with any publications that were generated from our research.

With respect to forming personal relationships, we leveraged person-centered recruitment approaches, which aim to appeal to individual's needs and priorities, as the first step to forming a trusting research relationship (26). For example, we used a combination of formal, scientific language to articulate the importance of the study and emotion-focused language to enhance feelings of personal connection during recruitment (24). This sensitivity is especially crucial for studies that consist of just a few encounters with researchers (27).

### Community-level barriers

Our team devised several strategies for overcoming community-level challenges to recruitment such as enrolling individuals from traditionally underrepresented groups, communicating with caregiver support groups, and navigating virtual recruitment.

#### Challenge: Accessing minority populations and rural communities

Inclusion of caregivers from traditionally underrepresented groups in research is foundational to enhancing the generalizability of study findings, given that one-third of the U.S. population identifies as a racial or ethnic minority (28) and almost one-fifth lives in a rural area (29). Older adults and caregivers in rural areas are particularly hard to reach as they rely on their local community networks for information and assistance (27, 30). With respect to racial and ethnic minorities, the historical discrimination of these groups in the healthcare system has resulted in a “recruitment crisis” whereby minority populations exercise caution in deciding to participate in research (31).

Another major challenge is internet inequity, which is associated with a variety of demographic variables including geographic region, socioeconomic status, gender, age, race, and ethnicity (32). Although social media platforms, such as Facebook, have been shown to be a useful research recruitment tool with benefits such as reduced costs, better representation, and increased access to hard-to-reach populations, the website's algorithm is not inclusive of the caregiver population (33, 34). Moreover, although online advertisements can be targeted to a certain age group or to persons with specific illnesses, caregiving is not included in these metrics (24). Lastly, limited availability of recruitment and research materials in languages other than English presented a barrier to recruiting and enrolling individuals from minority backgrounds.

#### Solutions

Following recommendations from prior literature (35, 36), we employed four strategies. First, we informed local stakeholders (e.g., community leaders, care coordinators) about the importance of our intervention, which helped to motivate personal referrals. Second, we provided potential participants with the opportunity to learn more about the study from a familiar source by facilitating connections between potential participants and physicians, educators, and religious leaders in the community who are willing to support research efforts.

Third, we appointed a co-investigator with strong community ties in rural areas to work with local organizations to encourage caregivers to participate in our study. Fourth, we utilized snowball sampling to elicit referrals from enrolled participants to other caregivers in their networks (13, 35, 36).

To minimize the impact of internet inequity, we recommend modifying the consent and recruitment procedures to reduce technological barriers for participants. For example, instead of video conferencing, we used more widely accessible modes of communication, such as phone calls, to facilitate correspondence.

To increase engagement of individuals from racial and ethnic minority groups, it is essential that researchers offer recruitment and research materials in languages other than English. While we did not provide these materials, we recommend that future researchers prioritize investing in hiring and training additional team members who are proficient in languages spoken by minority groups, collaborating with translators to prepare recruitment materials in other languages, and using translator services to screen non-English-speaking participants for eligibility *via* telephone can assist in improving the representativeness of samples.

#### Challenge: Accessing caregiver support groups

Whereas some support groups fall under a network with national reach, such as the Alzheimer's Association, others are geographically restricted. Below, we discuss differences and similarities we observed when recruiting from these two types of support groups.

A specific challenge to recruiting from local, small-scale support groups was that directors rarely provided the contact information for their clients. Instead, they offered our information to caregivers which placed the onus on the individual to contact the research team. In our experience, due to caregivers’ multiple responsibilities and time constraints, they were less likely to contact the research team even when eligible and interested. We also found that directors of local support groups are often caregivers themselves or lead their support group as a secondary role to their primary job. Thus, compared to leaders of national support groups, they are less familiar with research recruitment and require a more in-depth explanation of research studies.

Recruiting through large caregiver support groups sponsored by national organizations presents its own set of challenges. Larger organizations, such as the Alzheimer's Association, as well as 1,000 + member caregiver support groups on Facebook, often follow no-solicitation policies that ban caregiver support group leaders from sharing advertisements about research studies.

#### Solutions

To maximize our recruitment efforts, we tailored our recruitment strategies to the size and structure of each support group. When working with small-scale support groups, we leveraged principles from community-based participatory research to build trust and show our commitment to fostering a long-term community-research partnership (37). For example, we found that offering the intervention materials upon study completion to control group participants was an actionable way to incentivize directors to advertise the study because all parties stood to benefit. When recruiting from large support groups sponsored by national organizations, we offered recognition of their contributions by including their organization in the publication's acknowledgement section, and would recommend this strategy to future researchers (35, 38).

Irrespective of the size and scope of the support group, we found that citing our funding source (National Institute on Aging) and sharing the lead investigator's publications were simple and effective ways of legitimizing our research and improving recruitment. For both kinds of support groups, we requested directors to connect us directly with caregivers who may fit the inclusion criteria through an introductory email or phone call. If support group leaders are hesitant to make this introduction, future researchers can aid the director's comfort with the study team by listening to the director's concerns and making an in-person visit to establish a personal rapport if deemed feasible. If this does not increase director's comfort, we suggest leaving the research team's contact information with the director may be a better use of the team's time as they can focus on identifying additional support groups who may be more open to research.

### Institution-level barriers

We identified two elements of the research process at the institutional level that posed obstacles to recruiting family caregivers: Institutional Review Board (IRB) delays and challenges navigating academic research registries.

#### Challenge: Obtaining research consent

Gaining IRB approval is a necessary step for all studies involving participant involvement. A main concern of an institution's IRB is to ensure that all participants are informed of potential risks and benefits of the study prior to enrollment. This process traditionally involves reviewing an informed consent document with potential participants that details the background, involvements, funding, potential risks and benefits, and other key components of the study. However, a lengthy and technical consent process can limit enrollment of individuals, especially those who belong to ethnic minority or low socioeconomic status groups (39). The traditional in-person consent process also limits recruitment to individuals who are in close geographic proximity since they must be physically present to review and sign the consent document.

#### Solutions

We modified our study protocols to minimize the technical barriers to consenting and enrolling participants. To accommodate participants who were unable to consent in a traditional format, we utilized oral consent. This method decreases participant burden by eliminating the need to download, print, and scan a consent form, which is difficult for many older caregivers, and can lead to slower enrollment and more dropouts.

Our institutional IRB was hesitant to approve oral consent out of concern that it would lead participants to have an inadequate understanding of the study before enrolling. We had frequent conversations with staff from the IRB at our institution to develop the most appropriate, effective consent protocol for our study. Future researchers can further legitimize the oral consent process by appointing a research team member who is experienced liaising with participants and trained in research ethics, consent, and IRB requirements (38) and appointing at least one professional with expertise in geriatric medicine and/or behavioral research to sit on these boards (40).

#### Challenge: Identifying caregivers *via* research registries

The rise in online research registries renders them a useful tool for connecting researchers with people interested in participating in research. Most research registries are affiliated with academic institutions; however, there also exist trustworthy, independent, non-profit registries such as researchmatch.org, which is funded by the NIH. Interested volunteers can provide their contact information and basic health and demographic information to a research registry, and then may be invited by researchers to participate in specific studies.

We used two forms of research registries: (1) databases of interested individuals with specified diagnoses and (2) web-based platforms that connect interested individuals with relevant health studies. The first type of registry is a database of individuals with specific health conditions who provide their information and agree to be contacted by researchers. This first variation is best used for studies aiming to recruit participants with chronic conditions as significant time may pass between when a participant signs up for the registry and when they are matched with a research study. The second type of registry is structured such that ongoing research studies can be posted on a website where potential participants can browse through them and contact the research team if interested.

The second type of registry presented challenges in terms of building trust with caregivers since our study was one of many listed on a given research registry. We had greater success with local and institution-specific registries that connected interested individuals with relevant studies. Such registries send study information to potentially interested volunteers, and then volunteers can take the initiative of contacting researchers.

#### Solutions

We utilized and would recommend institution- or county-specific registries due to the added layer of familiarity and trust towards local institutions. For example, the Seniors Together in Aging Research (STAR), a database of Iowans over the age of 50 or are interested in volunteering for research studies, was a successful avenue for us in recruiting family caregivers. Moreover, volunteers on these registries have already demonstrated initiative and general knowledge of the demands of research participation. Also, we employed and would recommend research registries in which participants offer their contact information to researchers, or that directly connect participants with relevant studies, as an effective method to identify caregivers who are eligible and likely to participate in studies involving caregivers of PWD.

## Conclusion

Family caregivers of PWD play a crucial role in supporting their care recipient's personal, emotional, and healthcare needs. Yet, the realities of caring for a person with dementia place great strain on the caregiver's own well-being (41, 42). Thus, developing and evaluating interventions to support this vital, unpaid workforce is imperative.

The lessons learned from our pilot RCT can provide a roadmap for improving recruitment in future research initiatives. Table 1 summarizes barriers to recruitment and suggested solutions to enhance recruitment outcomes. At the individual level, building rapport and maintaining flexibility with potential participants was crucial for increasing caregiver motivation to participate and enrolling participants from a range of backgrounds. At the community level, building trust by collaborating with support group leaders, working with a co-investigator who was embedded in the local community, establishing equitable partnerships with target organizations, and utilizing technology in a way that minimized the impact of internet inequity to reach a broader range of caregivers all helped to overcome recruitment barriers.

**Table 1:** Summary of recruitment barriers and solutions.

Domain	Barriers	Solutions
**Individual level**	Working with caregivers’ time constraints	Use concise, simple language in correspondence with study participants Design study materials that are easy to read and direct Accommodate each participant’s preference regarding timing and mode of data collection
	Motivating caregivers to participate	Clarify possible benefits of participation, such as receiving education on caregiving Emphasize how their participation may ultimately help future caregivers Establish rapport with caregivers through compassionate and respectful interactions
**Community level**	Accessing minority populations and rural communities	Engage community figures and healthcare staff in recruitment efforts and ask for referrals to potential participants Facilitate conversations about the study between caregivers and community stakeholders Ask enrolled participants to refer other caregivers in their network
	Accessing caregiver support groups	Tailor recruitment strategies according to the size and structure of caregiver support groups Offer recognition of contribution in future publications Legitimize the study by sharing funding sources and prior publications from the research team
**Institutional level**	Obtaining research consent	Design study protocol to minimize technical barriers, such as using oral consent Train research staff in communication with participants, IRB requirements, and consent procedures Appoint a geriatric medicine and/or behavioral research professional to engage with the IRB
	Identifying interested caregivers *via* research registries	Use institution- or county-specific registries that offer greater familiarity and trust to participants Utilize registries in which researchers can contact potential participants or registries that connect participants to relevant studies

Once connected with communities, establishing research credibility is crucial. At the institutional level, collaborating with institutional IRBs and integrating the perspectives of experts in regulatory affairs and geriatric medicine or research would help overcome barriers in obtaining IRB approval while maintaining the quality of the consent procedure. Overall, improving strategies to optimize recruitment of caregivers of PWD into clinical research studies is a critical step towards identifying evidence-based practices to aid this “unseen” labor force.

## Data Availability

The raw data supporting the conclusions of this article will be made available by the authors, without undue reservation.
